# A literature review for the mechanisms of stress‐induced liver injury

**DOI:** 10.1002/brb3.1235

**Published:** 2019-02-13

**Authors:** Jin‐Yong Joung, Jung‐Hyo Cho, Yun‐Hee Kim, Seung‐Hoon Choi, Chang‐Gue Son

**Affiliations:** ^1^ Liver and Immunology Research Center Daejeon Oriental Hospital of Daejeon University Daejeon Korea; ^2^ Korean Medicine Convergence Research Division Korea Institute of Oriental Medicine (KIOM) Daejeon Korea; ^3^ Department of Life Convergence Graduate School of Dankook University Yongin Korea

**Keywords:** hepatic immune tolerance, hypoxia‐reoxygenation, liver injury, oxidative stress, stress

## Abstract

**Introduction:**

Experimental studies and clinical observations have shown that stress can damage hepatic tissue both directly and indirectly. Many studies have partially revealed the contributors of stress‐induced liver injury; however, the whole process has not yet been uncovered. This review aims to summarize the mechanisms that have been proposed to be involved.

**Methods:**

A literature search was conducted using PubMed (http://www.ncbi.nlm.nih.gov/pubmed) in its entirety up to March 2018, and analyzed the animal‐derived mechanistic studies on stress‐induced liver injury.

**Results:**

The liver is the organ that meets and filters a mass of alien material, and then maintains immune tolerance under physiological conditions. Under stress conditions, however, immune tolerance is interrupted, which results in the induction of inflammation in the liver. Contributors to this process can be categorized as follows: hypoxia‐reoxygenation, over‐activation of Kupffer cells and oxidative stress, influx of gut‐derived lipopolysaccharide and norepinephrine, and over‐production of stress hormones and activation of the sympathetic nerve.

**Conclusions:**

Psychological stress is associated with a variety of pathological conditions resulting in liver injury through multiple systems, including the sympathetic nervous and adrenocortical system. Mechanistic understanding of this phenomenon is important for the clinical practice of managing patients with hepatic disorders and should be explored further in the future.

## 1 INTRODUCTION

Stress refers to internal state of threatened homeostasis caused by threat or challenge to the organism's well‐being (Ulrich‐Lai & Herman, 2009). In recent stress studies, the term stress is often restricted to “conditions where an environmental demand exceeds the natural regulatory capacity of an organism, in particular situations that include unpredictability and uncontrollability” (Koolhaas et al., 2011). Stress responses cause pathological conditions through multiple systems, including the sympathetic nervous and adrenocortical system (Goldstein & Kopin, 2007). Stress negatively influences not only the development but also the progression of various disorders, such as cardiovascular, psychiatric, endocrine, and cancerous disorders (Cohen, Janicki‐Deverts, & Miller, 2007; Reiche, Nunes, & Morimoto, 2004).

On the other hand, liver receives blood from the hepatic artery and the portal vein. In human, former and latter contribute 20%–25% and 75%–80% of the blood, respectively (Rappaport, 1980). The portal vein in particular delivers alien substances and antigens, such as digested products, old or damaged cells, and microbial products, to the liver, which may result in a high risk of excessive immune activation (Racanelli & Rehermann, 2006). Under physiological conditions, the liver ignores the immune challenge of various antigens, which is termed liver immune tolerance (Tiegs & Lohse, 2010). However, this immune tolerance process can be distorted by any pathological circumstance including psychological stress (Liu, Wang, & Jiang, 2017).

An episode of stress has also been recognized to worsen clinical symptoms and hepatic biochemistry parameters in patients with chronic type B or C viral hepatitis (Kunkel et al., 2000; Nagano, Nagase, Sudo, & Kubo, 2004), alcoholic hepatitis (Fukudo, Suzuki, Tanaka, Iwahashi, & Nomura, 1989), nonalcoholic steatohepatitis (Elwing, Lustman, Wang, & Clouse, 2006), and autoimmune hepatitis (Srivastava & Boyer, 2010). In addition, many animal studies have provided evidence that restraint stress and electric foot shock stress are the direct causes of liver injury (Chida, Sudo, Motomura, & Kubo, 2004; Fernández et al., 2000; Tseilikman et al., 2012; Zhu et al., 2014), and foot shock stress exaggerates hepatotoxicity in a chemical‐induced mouse model (Shimazu, 1986). These liver injuries were observed as substantial damage of the hepatocytes in the form of elevated liver enzymes and liver histologic abnormalities. In current society, stress is an inevitable factor, and evidence for a link between the brain and the liver has accumulated (Campbell et al., 2003; D'Mello & Swain, 2011).

Liver injury (i.e., substantial damage of hepatocytes) can occur only by stress without other medical conditions; however, little is known regarding the underlying mechanisms. In this study, we aimed to provide an overview of the corresponding mechanisms for stress‐induced liver injury from the animal studies conducted to date.

## 2 METHODS

### 2.1 Data collection

In order to collect data on mechanisms of stress‐induced liver injury, a literature search was conducted using PubMed (http://www.ncbi.nlm.nih.gov/pubmed) in its entirety up to March 2018. These searches were performed using combinations of the following keywords: “stress”, “liver injury”, “liver damage”, and “hepatitis” respectively. Additional survey was performed by screening references of the selected articles. References of these additional investigated articles have also been researched for their usefulness.

### 2.2 Data analysis

Three hundred and seventy‐four articles at initial screen, twenty‐five papers were finally selected, which contained any mechanistic information of stress‐induced liver injury by mainly experimental researches. Authors have reviewed carefully the contents, and classified those findings.

## 3 RESULTS

### 3.1 Features of the liver immune tolerance

The most well‐known mechanism of immune tolerance of the liver is the prominent secretion of interleukin 10 (IL‐10) by Kupffer cells. Under physiological levels of lipopolysaccharide (LPS) from the intestinal canal, Kupffer cells consistently secrete IL‐10 to suppress the activation of other immune cells and the production of proinflammatory cytokines (Knolle & Gerken, 2000). The secretion of IL‐10 by Kupffer cells occurs rapidly within 2 hr of LPS stimulation, contrary to other monocytes that secrete IL‐10 within 24 hr, only as a compensatory reaction (Kamei, Callery, & Flye, 1990; Knolle et al., 1998). The key role of Kupffer cells in hepatic tolerance was demonstrated by animal experiments with the depletion of Kupffer cells by chemical compounds, in which immunologic tolerance disappeared (Kamei et al., 1990; Roland, Mangino, Duffy, & Flye, 1993).

Kupffer cells have various toll‐like receptors (TLRs). Among them, TLR3 can be activated by other antigens, including necrotic debris or double stranded Ribonucleic Acid (dsRNA) of a virus, which leads to IL‐18 production via the TIR–domain containing adapter‐inducing interferon‐β (TRIF) pathway in Kupffer cells and the sequential activation of natural killer cells (NK cells) (Tu et al., 2008). Interferon gamma (IFN‐γ) secreted by activated NK cells induces the polarization of Kupffer cells into M1 macrophages that produce proinflammatory cytokines (Chávez‐Galán, Olleros, Vesin, & Garcia, 2015). Under general circumstances, however, physiological levels of LPS bind TLR4 on Kupffer cells and consequently produce IL‐10, inhibiting the activation of hepatic immune cells via the myeloid differentiation primary‐response gene 88 (Myd88) pathway, referred to as M2 macrophage polarization (Biswas & Mantovani, 2010; Martinez & Gordon, 2014).

### 3.2 Major contributors to liver injury under stress conditions

#### 3.2.1 Hypoxia‐reoxygenation

Stress leads to the reduction in hepatic blood flow, which is mediated by the hypothalamus–hepatic sympathetic nerve–norepinephrine axis and hypothalamus–adrenal medulla–epinephrine axis (Chida, Sudo, & Kubo, 2006). Using rat experimental models, Chida Y *et al.* showed a 60% reduction in hepatic blood flow after exposure to electric foot shock or psychological stress (Chida, Sudo, & Kubo, 2005). Hypoxia in hepatic tissue causes the production of reactive oxygen species (ROS), mainly in the mitochondria, leading to endoplasmic reticulum stress and to the necrosis of cells (Chandel et al., 2000; Xu, 2005). In addition, upon reperfusion of the blood flow, Kupffer cells and endothelial cells are activated to produce ROS and to secrete various inflammatory cytokines (Carden & Granger, 2000; Teoh & Farrell, 2003). These blood flow alterations involve the secretion of corticotropin‐releasing factor (CRF) from the hypothalamus. From Chida Y's acute stress mouse experiment, the reduction in hepatic blood flow was significantly ameliorated by an injection of the CRF receptor antagonist (Chida et al., 2005). Yoneda M *et al.* found that the central administration of CRF inhibited hepatic blood circulation through sympathetic and noradrenergic nerve pathways and proposed CRF as a neurotransmitter regulating hepatic blood flow in the central nervous system (Yoneda et al., 2005). In a chronic immobilization stress model by Bonaz B *et al*., the hepatic blood flow was not observed, but the CRF and CRF receptor activity in the chronic stress condition were less than that under acute stress (Bonaz & Rivest, 1998). This result suggests that blood flow alteration due to stress is more noticeable under acute stress rather than chronic stress conditions through the over‐secretion of CRF.

The hypoxia‐reperfusion damage in liver mainly causes necrosis of hepatocytes rather than apoptosis. Using a partial ischemia rat model, Gujral JS *et al.* found that the major injury is initiated by necrosis in hepatocytes and sinusoidal epithelial cells after reperfusion, and a small minority of those cells undergo apoptosis (Gujral, Bucci, Farhood, & Jaeschke, 2001). The hypoxic condition is unable to produce oxidative phosphorylation, resulting in a deficiency in adenosine triphosphate (ATP) production; then, this insufficient ATP supply generally shifts apoptosis into necrosis of hepatocytes. If blood is reperfused in the ischemic hepatic tissues, and the ATP level is partially restored, hepatic necrosis is switched to apoptosis, and the hepatic inflammation gradually lessens (Guicciardi, Malhi, Mott, & Gores, 2013). Actually, low level of ATP exacerbate ischemia‐reperfusion injury in human hepatic transplantation (Lanir et al., 1988). Under stress conditions, reduced hepatic ATP levels have been shown in both rats and mice (Berglund et al., 2009; Bravo, Vargas‐Suárez, Rodríguez‐Enríquez, Loza‐Tavera, & Moreno‐Sánchez, 2001). This stress‐induced depletion of ATP would cause necrosis rather than apoptosis, leading to more severe liver injury. In addition, hypoxic conditions release various tissue‐derived inflammatory mediators such as endothelial monocyte‐activating polypeptide II, endotheline, vascular endothelial growth factor, and mitogen‐activated protein kinase phosphatase‐1, which is followed by the recruitment of macrophages resulting in inflammatory injury (Chanmee, Ontong, Konno, & Itano, 2014).

#### 3.2.2 Over‐activation of Kupffer cells and oxidative stress

Under stress conditions, the decrease in hepatic blood flow induces mitochondrial hypoxia, which activates the production of ROS, resulting in cell necrosis. Imbalance between oxidative stressors and antioxidant components, called as oxidative stress, is known to be an initiator or common mediator under various hepatic damages including stress‐induced liver injury (Tseilikman et al., 2012). Oxidative stress can also lead to the over‐production of proinflammatory cytokines that induce the infiltration and activation of inflammatory cells such as neutrophils, monocytes, and lymphocytes. These activated inflammatory cells produce more ROS, which exacerbates the oxidative stress as well as triggering inflammation and hepatic necrosis (Mittal, Siddiqui, Tran, Reddy, & Malik, 2014).

The necrosis of liver cells also initiates the progression of liver injury, which provides a signal to the surrounding tissue, especially immune cells (Kono & Rock, 2008). Necrotic cells in the hepatic tissue leak damage/danger‐associated molecular patterns (DAMPs). DAMPs, such as high mobility group box 1 (HMGB), messenger RNA (mRNA), heat shock proteins and IL‐1α, are derived from hosts and are unrelated to the pathogen. DAMPs are generally hidden within host cells and are ignored by the immune system, but when released outside of the cell, they activate the immune system and induce inflammation (Kubes & Mehal, 2012). In particular, the IL‐1α, one of the DAMPs, stimulates Kupffer cells to produce both IL‐1α and IL‐1β in large amounts through the NF‐κB pathway (Dinarello, 2011). The IL‐1α and IL‐1β act on hepatocytes to induce the secretion of chemokine (C‐X‐C motif) ligand 1 (CXCL1), causing the infiltration of neutrophils around the hypoxic region (Chen & Nuñez, 2010; Rider et al., 2011). Kupffer cell‐derived IL‐1 has been known to play a key role in the production of ROS and TNF‐α by neutrophils. From two ischemia‐reperfusion rat models by Shirasugi N's and Shito M's groups, preinjection with an IL‐1 receptor antagonist blocked the production of endogenous IL‐1 and ROS production, and protected the liver tissue along with reducing leukocyte attachment to endothelial cells and the tumor necrosis factor‐α (TNF‐α) level in the hepatic tissue (Shirasugi et al., 1997; Shito et al., 1997). Another mouse restraint stress experiment by Tseilikman V *et al.* showed that pretreatment with an IL‐1 antagonist reduced liver injury as demonstrated by significantly lower levels of hepatic enzymes and infiltrated immune cells (Tseilikman et al., 2012).

In addition, the mRNA that leaks from necrotic cells activates the TLR3 pathway in Kupffer cells, which leads to IL‐18 secretion by Kupffer cells and sequentially activates NK cells to produce IFN‐γ (Karikó, Ni, Capodici, Lamphier, & Weissman, 2004; Tu et al., 2008). The IFN‐γ, with or without LPS, differentiates Kupffer cells into M1 macrophages that accelerate the inflammatory reaction in hepatic tissue. When Kupffer cells are polarized to M1, immunologic tolerance in the liver is disrupted through the imbalanced secretion of anti‐ and proinflammatory cytokines, with IL‐10 likely secreted in small amounts but IL‐12 secreted in large amounts (Martinez & Gordon, 2014). IL‐12 is a proinflammatory cytokine that stimulates T cells and other lymphocytes. Overproduction of IL‐12 activates hepatic Th1 cells to secrete both IFN‐γ and TNF‐α, which stimulates Kupffer cells and induces inflammation (Hammerich & Tacke, 2014). In contrast, IL‐10 is a major anti‐inflammatory cytokine with important roles in protecting cells and organs by inhibiting excessive immune responses (Borish et al., 1996). Increased IL‐12 and decreased IL‐10 can cause the liver to become vulnerable to an inflammatory reaction.

#### 3.2.3 Influx of gut‐derived LPS and norepinephrine

Under stress conditions, hypothalamus‐secreted CRF, neuron‐released acetylcholine and glucocorticoids from the adrenal cortex increase the permeability of the intestinal canal and make the portal vein prone to an influx of LPS or other antigens (Castagliuolo et al., 1996; Meddings & Swain, 2000; Santos et al., 1999). In particular, CRF and acetylcholine mediate stress‐induced mast cell activation and ion secretion, leading to increases in intracellular permeability and transcellular uptake in the gastrointestinal tract (Söderholm & Perdue, 2001). Santos J *et al. *demonstrated that mast cell‐deficient mice do not display an increase in gut permeability under stress. Bioactive chemicals (not yet identified) secreted from mast cells are suggested to play a major role in increasing gut permeability (Santos, Benjamin, Yang, Prior, & Perdue, 2000). The stress‐induced increase in gut permeability has been observed in both humans and mice. In human, under public speech stress, gut permeability was approximately twice that of the control (Vanuytsel et al., 2014). In animal studies, the degree of the increase in gut permeability varied from 1.5‐ to 2‐fold depending on the intensity of the restraint stress (Santos et al., 1999; Saunders, Kosecka, McKay, & Perdue, 1994).

The above stress‐induced increase in gut permeability augments the influx of gut‐derived LPS and alien substances into the liver (Frazier, DiBaise, & McClain, 2011). Contrary to physiological levels of LPS, over‐influx of LPS from the gut shifts the polarization of Kupffer cells into M1 macrophages, leading to vulnerability of the liver to inflammation, and the increased levels of alien substances easily damage the liver (Li et al., 2015; Nguyen‐Lefebvre & Horuzsko, 2015). The role of gut LPS in hepatic inflammation has been supported by an ethanol‐induced hepatic injury experiment. Adachi Y *et al.* demonstrated a significant reduction in ethanol‐induced hepatic injury by administering antibiotics to deplete gut microbiota (Adachi, Moore, Bradford, Gao, & Thurman, 1995). Moreover, the mice injected with ethanol along with zinc (as an inhibitor of gut permeability) showed neither an increase of LPS in the blood nor liver injury (Lambert et al., 2003).

Psychological stress also decreases intestinal blood flow through a combination of sympathetic activation and parasympathetic suppression, as well as the release of stress hormones, such as neuropeptide Y (Matheson, Wilson, & Garrison, 2000). When the reduction and reperfusion of blood flow occurs in the gut, the intestine responds and secretes a high level of norepinephrine that enters the liver (Aneman, Medbak, Watson, & Haglind, 1996). Norepinephrine in liver can induce Kupffer cells to secrete TNF‐α at levels four to sevenfold that of controls, which is completely blocked by treatment with the α‐2 adrenergic antagonist yohimbine (Zhou et al., 2001). These results suggest the important roles of gut‐derived norepinephrine in stress‐related hepatic injury via activation of Kupffer cells to produce the inflammatory cytokine TNF‐α in the liver.

#### 3.2.4 Over‐production of stress hormones and activation of the sympathetic nerve

Stress provokes the activation of the HPA axis, leading to a release of stress hormones that are known as glucocorticoids. In a mouse restraint stress model, Nair A showed that serum corticosterone increased 3–5 times compared with that in the control (Nair & Bonneau, 2006). In humans, Rohleder N et al. reported that the cortisol level was increased twofold in saliva and 1.6‐fold in plasma in subjects stressed via tasks such as a free speech in front of an audience and a mental arithmetic task of 10‐min duration (Rohleder, Kudielka, Hellhammer, Wolf, & Kirschbaum, 2002). Glucocorticoids are known to generally play a role of immune suppressants (Coutinho & Chapman, 2011). However, some studies have reported the opposite actions of cortisol or corticosterone, especially on liver inflammation. There are clinical observations of hepatic injury after the administration of corticosteroid (D'Agnolo & Drenth, 2013; Gutkowski, Chwist, & Hartleb, 2011). An animal study has also shown glucocorticoid‐induced hepatotoxicity (Rogers & Ruebner, 1977). Moreover, Chensue SW et al. demonstrated an aggravation of liver damage and proinflammatory cytokines (IL‐6 and TNF‐α) by stress‐related doses of corticosterone in LPS‐challenged mice (Chensue, Terebuh, Remick, Scales, & Kunkel, 1991). However, the underlying mechanisms responsible for the proinflammatory action of glucocorticoids are still uncertain. One possibility follows. The stress hormones, particularly corticosterone, are known to distribute immune cells into inflammatory regions and to accelerate the initial inflammatory reaction. Using an adrenalectomy and corticosterone‐replacement rat model, Dhabhar FS et al. demonstrated that stress‐derived corticosterone induces the trafficking of leukocytes to battle stations in acute stress but not in chronic stress (Dhabhar, Malarkey, Neri, & McEwen, 2012).

Stress stimulates the sympathetic‐adrenal‐medullary (SAM) system to secrete the two catecholamines epinephrine and norepinephrine. Many studies have reported that catecholamines are involved in cytotoxicity and tissue damage, including in the liver. Epinephrine has been reported to promote the production of hydroxyl radicals in isolated rat hepatocytes (Yang et al., 2012). From animal experiments, norepinephrine administration has been shown to increase the production of two proinflammatory cytokines, TNF‐α and IL‐6, by Kupffer cells and hepatocytes, respectively (Jung et al., 2000; Zhou et al., 2001). Surgical and chemical hepatic sympathectomy notably inhibited the restraint stress‐induced production of IL‐6 in hepatic tissue (Nukina et al., 2001; Takaki, [Link]; Takaki, Huang, Somogyvári‐Vigh, & Arimura, 1994). Zhu Q et al. also showed the important action of catecholamines on stress‐induced hepatic apoptosis using catecholamine receptor antagonists, where α‐1 and α‐2 (but not β‐1 and β‐2) adrenoreceptor antagonists resisted liver injury under restraint stress (Zhu et al., 2014).

## 4 CONCLUSION

From the experimental data described above, we can summarize the underlying mechanisms for stress‐induced liver injury as follows. Stress conditions can induce hepatic hypoxia and reperfusion, the over‐influx of gut LPS, and an increase in the release of stress hormones, including corticosteroids and catecholamines. This systemic alteration of the hepato‐intestinal blood flow first causes the partial necrosis of hepatocytes, which can leak intracellular substances. The leakage of unwanted antigens, called DAMPs, initiates the inflammatory reaction surrounding the hypoxic region and consequently activates NK cells to produce IFN‐γ, leading to M1 polarization of Kupffer cells.

**Figure 1:**
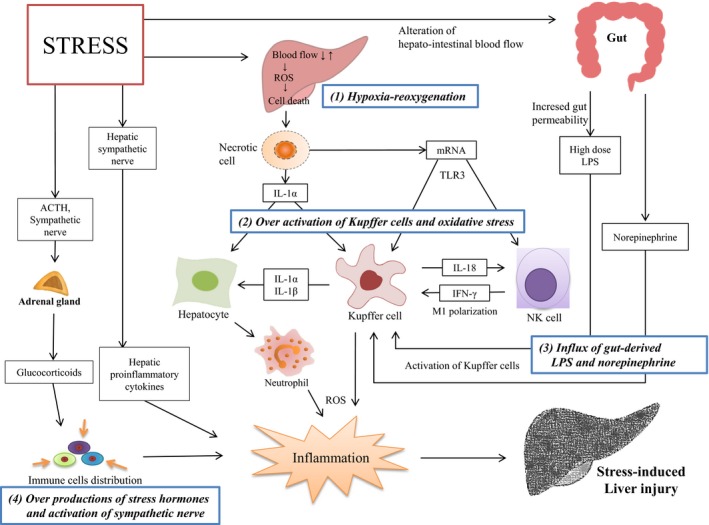
Summary of stress‐induced liver injury. SAM: sympathetic‐adrenal‐medullary, HPA: hypothalamic‐pituitary‐adrenal, ROS: reactive oxygen species, LPS: lipopolysaccharide, IFN‐γ: interferon gamma, IL: interleukin

This condition indicates the break of the tolerance of Kupffer cells, which is accelerated by the over‐influx of gut LPS and norepinephrine. Over activation of Kupffer cells begins to substantially provoke the inflammatory environment in hepatic tissue, mainly via the production of IL‐1 and ROS with other cells, including immigrated neutrophils. Moreover, the stress‐related over‐release of corticosteroids and catecholamines can promote the further migration of peripheral leukocytes into hepatic tissue and the production of inflammatory cytokines such as TNF‐α and IL‐6. The activation of the sympathetic nerve may also augment the susceptibility of hepatic tissue to stress‐related inflammation. This study is summarized in Figure 1.

The present review has limitations, especially its reliance on results from mainly animal studies. Moreover, there is still insufficient evidence to explain the mechanisms responsible for stress‐associated hepatic damage. Further researches are warranted from both human studies and animal models. Prospective clinical studies could be helpful, for example such as demonstrating a positive correlation between psychological stress index and hepatic biochemistry parameters. The adaptations of animal models with adrenalectomy, germ‐free guts or depletion of NK cells or Kupffer cells are recommended to explore these areas in the future. In addition, there should be consideration regarding the intensity and mode of the stress period on the risk of hepatic damage.

The psychiatric influence of stress is garnering more attention in medical practice. Clinical cases with elevated liver enzymes without any known causes are often observed. Obviously, stress response could be a possible reason, which has been overlooked so far. We hope that the present review boosts future studies addressing the entire molecular mechanisms for the stress‐induced hepatic damage or stress‐associated influence on liver disorders.

## CONFLICTS OF INTEREST

There are no conflicts of interests.
